# Clinical and Immunological Effects of rhIL-2 Therapy in Eastern Chinese Patients with Multidrug-resistant Tuberculosis

**DOI:** 10.1038/s41598-017-18200-5

**Published:** 2017-12-19

**Authors:** Qi Tan, Rui Min, Guan-qun Dai, Yan-li Wang, Li Nan, Zhen Yang, Jun Xia, Shi-yang Pan, Huang Mao, Wei-ping Xie, Hong Wang

**Affiliations:** 1 Department of Respiratory and Critical Care Medicine, the First Affiliated Hospital of Nanjing Medical University, Nanjing, 210029 China; 2 Jiangbei Hospital, Nanjing, 210029 China; 3 Jiangsu Province Hospital of TCM, the Affiliated Hospital of Nanjing University of TCM, Nanjing, 210029 China; 4 Department of Laboratory Medicine, the First Affiliated Hospital of Nanjing Medical University, Nanjing, China

## Abstract

It is urgent to find an optimised therapy regimen for the control of MDR-TB globally. This study aimed to evaluate the efficiacy and safety of a combined regimen of rhIL-2 injection and standard chemotherapy within 18-month duration in a randomized controlled trial conducted in 14 centres in eastern China. From Jan. 2009 to July. 2016, 271 MDR-TB cases were enrolled and followed up in two groups, 142 cases in study group while 129 cases in control group. Clinical efficacy, safety and immune activity (Th1, Th17, Treg, IFN-γ, IL-17) among the two groups were evaluated and compared. After 24-month following up, cure rate in IL-2 group show higher than that in control group (56% VS 36%, P < 0.01). Rate of mycobacterium clearance (sputum negative) within 3 months was significantly higher in IL-2 group (74% VS 59%, P < 0.05) with no adverse events raised. Patients after rhIL-2 treatment showed increasing of Th1 populations and decreasing of Th17 and Regulatory T cells (Treg) populations, while levels of IL-17A, ROR-γt, and Foxp3 mRNA decreased and level of IFN-γ mRNA increased in PBMCs. Thus, rhIL-2 combined regimen within shorter duration achieved high conversion and success rates and improved Th1/Th17 immune responses, with no safety concerns emerging in MDR-TB patients.

## Introduction

Multidrug-resistant tuberculosis (MDR-TB), defined as resistance to at least isoniazid and rifampin, has emerged as a lethal global threat, according to a 2009 WHO report^1^. The treatment for MDR-TB often shows higher rates of treatment failure and deaths than that for drug-sensitive tuberculosis (DS-TB)^2^. As current second-line drugs are more toxic, require 24-month or prolonged regimens with daily administration, and a high cost, the rational design of a new treatment regimen that shortens the therapeutic period and provides a more efficacious treatment for MDR-TB is urgently required^3,4^.

IL-2 is a pleiotropic cytokine that is produced after antigen activation and plays crucial roles in the immune response^5–8^. IL-2 therapy regimens were expected to restore the immune response or to change the immunologic status^6,7^, thus allowing the host to more efficiently contain and eradicate immune responses, primarily those against cancers and infectious diseases^9–11^. Our previous finding has demonstrated the beneficial effect of rhIL-2 in 50 patients who enrolled before 2011.July, with limited follow-up over 12 months^12^. Moreover, in our previous study, patients with MDR-TB performed more distinctly depressed Th1 population, enriched Th17 population and Treg population than patients with DS-TB, in compare to healthy controls^13–15^.

Th1 cells are widely known to be key players in promoting the immune response associated with TB^16,17^. T helper 17 (Th17) cells, which are characterized by their expression of pro-inflammatory cytokines, such as IL-17A, IL-17F, IL-6, associated to promote granulomatous inflammation^18,19^. Recent studies have demonstrated, the balance between Th17-mediated protection and pathology is crucial for determining the outcome of infections at the mucosa and other organs^20–22^. We hypothesized that IL-2 play a crucial role in modulating Th17, Treg cells responses in patients with MDR-TB, thereby maintaining the balance between protection and pathology, which defines the outcome of MDR-TB infections. Until now, large studies to evaluated efficacy and safety of an immunomodulator treatment for MDR-TB are lacking. Outcome of a combined therapy with IL-2 agent during and after long-course follow-up has not previously been measured on a large population cohort.

To address this, in coordination by the network centers under the Center for Disease Control (CDC) of Jiangsu Province for TB control we conducted a prospective randomized controlled multicenter cohort study on 8-month adjunctive immunotherapy with rhIL-2within a background regimen (as per WHO guidelines) when treating MDR-TB cases.

The aim of the present study is to evaluate the safety, tolerability and effectiveness of the novel rhIL-2 within background regimens in a large multicentre cohort of MDR-TB patients treated under two treatment arms (rhIL-2 within chemotherapy regimen vs. chemotherapy regimen). We also first launched the present pilot study by investigating the kinetics of the activation of Th1, Treg, and Th17 cells from these patients in different stages of regimen by flow cytometry and evaluating the mRNA levels of their homologous cytokines by qRT-PCR as immune parameters to shed light on the mechanisms underlying the beneficial effect of rhIL-2 immunotherapy which still remained incompletely understood.

## Results

### Study population

**Figure 1:**
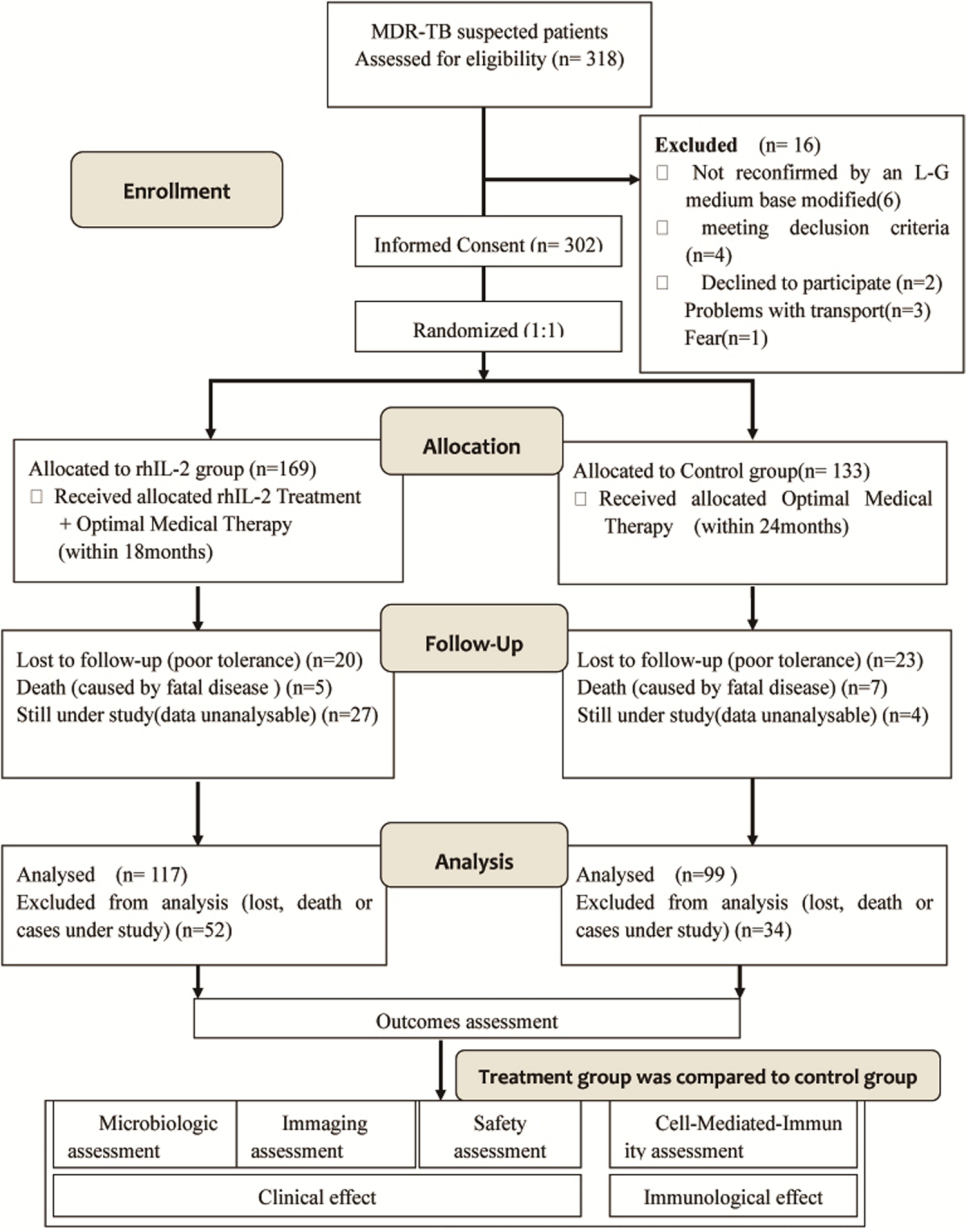
Flow diagram of the enrollment, follow-up and analysis of study participants. Totaly 16 patients discontinued the study before randomization, 302 patients were enrolled and randomly assigned to two study groups, 117 valid cases in rhIL-2 group and 99 valid cases in control group were included in the analysis. To observe clinical effect of two groups, microbiologic assessment, immaging assessment and safety assessment were performed during follow-up among 302 patients enrolled. To observe immune effect T cell status were measured in 25 patients in rhIL-2 group and 25 patients in control group, who were randomly selected from two groups. Outcomes were evaluated and compared among all of the valid cases of two groups.

The screening period began on July 1, 2009 and the last treatment visit of the last patient was on July 30, 2016. The patient selection was show in flowchart (Fig. 1). Cases were enrolled in two cohorts respectively from 14 sub-centers of Jiangsu Province around eastern China, details see Supplementary Table S1. The demographic and baseline characteristics were similar between the 2 study groups (Table 1).

**Table 1:** Baseline characteristics of two cohorts.

	**Control group**	**RhIL-2 group**	**Homogeneity of variance test P value**	**Independent t test/ Chi-square test P value**
Enrollment cases	133	169	/	/
Valid cases	99 (76.7%)	117 (82%)	/	0.38
Age	44.56 ± 14.18	43.89 ± 14.53	0.51	0.77
Male/Female	75/58	93/76	/	0.90
Relapsing case	56/64 (86%)	58/60 (91%)	/	0.12
Past history of treatment times(median)	2.85 ± 0.81	2.67 ± 0.73	0.23	0.31
Number of resistant anti-tuberculosis drugs(median)	2.90 ± 0.92	2.93 ± 0.82	0.44	0.47
Number of lung cavitation bilaterally	1.51 ± 1.09	1.70 ± 0.98	0.40	0.38
Number of lung fields with lesions	4.13 ± 1.75	4.17 ± 1.60	0.55	0.72

A total of 302 cases were enrolled in study with 169 cases in control group and 133 cases in rhIL-2 group. The median age of patients, ratio of male to female, relapsing cases, past history of treatment times, number of resistant anti-tuberculosis drugs, and radiographic findings at baseline were similar between the two groups (*p* > 0.05).

### Safety

Safety and tolerable information on both regimens were evaluated. No severe allergic reactions occurred during or after subcutaneous administration of rhIL-2 (0–8 months). Several isolated cases in the rhIL-2 group developed local pain and itchy skin. No Adverse events occurred in both groups. All types of adverse reactions occurred in follow-up duration (0–24 month) were similar between the two groups (P > 0.05), incidence rates compared and summarized in Table 2.

**Table 2:** Incidence of adverse events (AE) from chemotherapeutics for optimised standard anti-MDR-TB drugs among patients.

Adverse Reactions	**RhIL-2 Group N = 117**	**Control Group N = 99**	**P value**
Hepatic toxicity	13 (11.1%)	29 (29.3%)	0.55
Renal toxicity	8 (6.8%)	4 (4.0%)	0.55
Gastrointestinal trouble	20 (17.1%)	20 (20.2%)	0.68
Bone marrow toxicity	5 (4.3%)	3 (3.0%)	0.9
Ototoxicity	1 (0.9%)	3 (3.0%)	0.49
Eye toxicity	0	1 (1.0%)	0.93
Allergic reaction	3 (2.6%)	3 (3.0%)	0.83
Neuropsychiatric symptoms	2 (1.7%)	1 (1.0%)	0.88
Musculoskeletal pain	16 (13.7%)	13 (13.1%)	0.93
Electrolyte level abnormalities	10 (8.5%)	12 (12.1%)	0.52
Thyroid dysfunction	5 (4.3%)	9 (9.1%)	0.24

The adverse events include those reported by at least two patients in either treatment group during the treatment period, regardless of severity or causality. Incidences of 11 different, common adverse drug-reactions during the standard drug regimen were counted and compared between the rhIL-2 treatment group and the control treatment group. The data was analyzed by chi–square test or fish’s exact test, with no statistical differences found for any of the adverse reactions (*P value* > 0.05).

### Primary outcome

**Figure 2:**
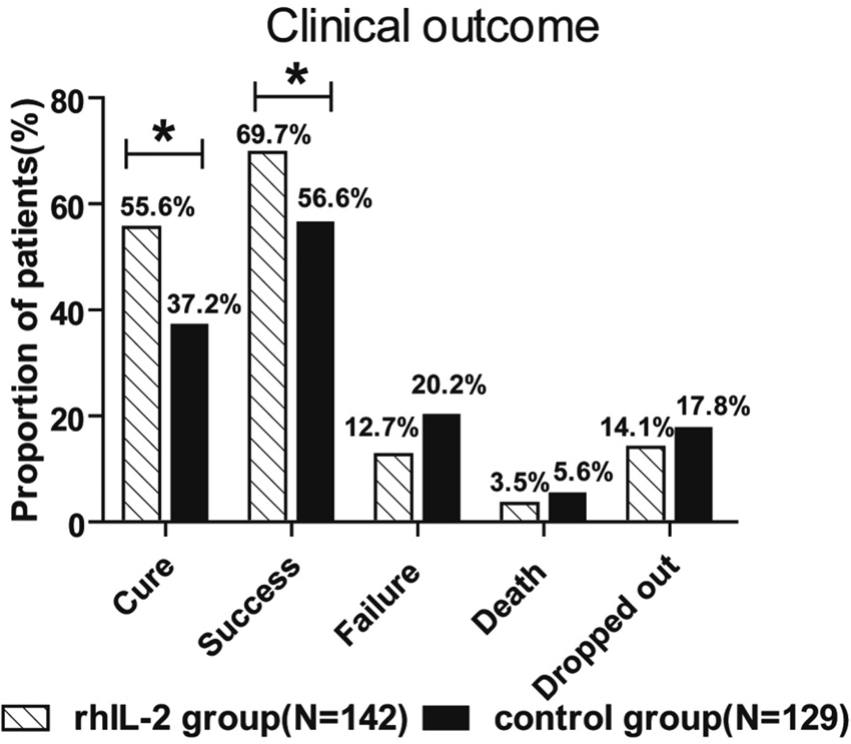
Outcome of the rhIL-2 group over an 18-month time course and the control group over a 24-month time course. Among 142 MDR-TB patients enrolled in the rhIL-2 treatment group, 55.6% were cured, 69.7% showed treatment success 79 cases cured, 20 cases completing treatment, 12.7% showed treatment failure, 3.5% died, and 14.1% dropped out. Among 129 MDR-TB patients enrolled in the control treatment group, 37.2% were cured, 56.6% showed treatment success, 20.2% showed treatment failure, 5.4% died, and 17.8% dropped out. The patients in the rhIL-2 treatment group had a significantly higher cure rate (55.6%) than those in the control treatment group (37.2%; *p* = 0.003) (*p < 0.05) and higher success rate (69.7%) than those in the control treatment group (56.6%; *p* = 0.034) (*p < 0.05).

Of 302 participants enrolled, 216 completed treatment and following up, 12 died, 43 defaulted, 31 ongoing (unable to be evaluated). Out of 142 evaluated cases in rhIL-2 group, 79(55.6%) were cured, 99(69.7%) achieved success (79 cured and 20 completing treatment), 18(12.7%) failed, 5(3.5%) died, and 20(14.1%) defaulted. Out of 129 evaluated cases in control group, 48(37.2%) were cured, 73(56.6%) achieved success (48 cured and 25 completing treatment), 26(20.2%) failed, 7(5.4%) died, and 23(17.8%) defaulted. The treatment success rate and cure rate in rhIL-2 group distinctly preceded that in control group (69.7% VS. 56.6%, 55.6% VS. 37.2%, *p* = 0.034, *p* = 0.003, two-tailed), while the failure rate and death rate were similar between two groups (*p* = 0.13, *p* = 0.64, two-tailed) (Fig. 2, Supplementary Table S2).

### Secondary outcome

**Figure 3:**
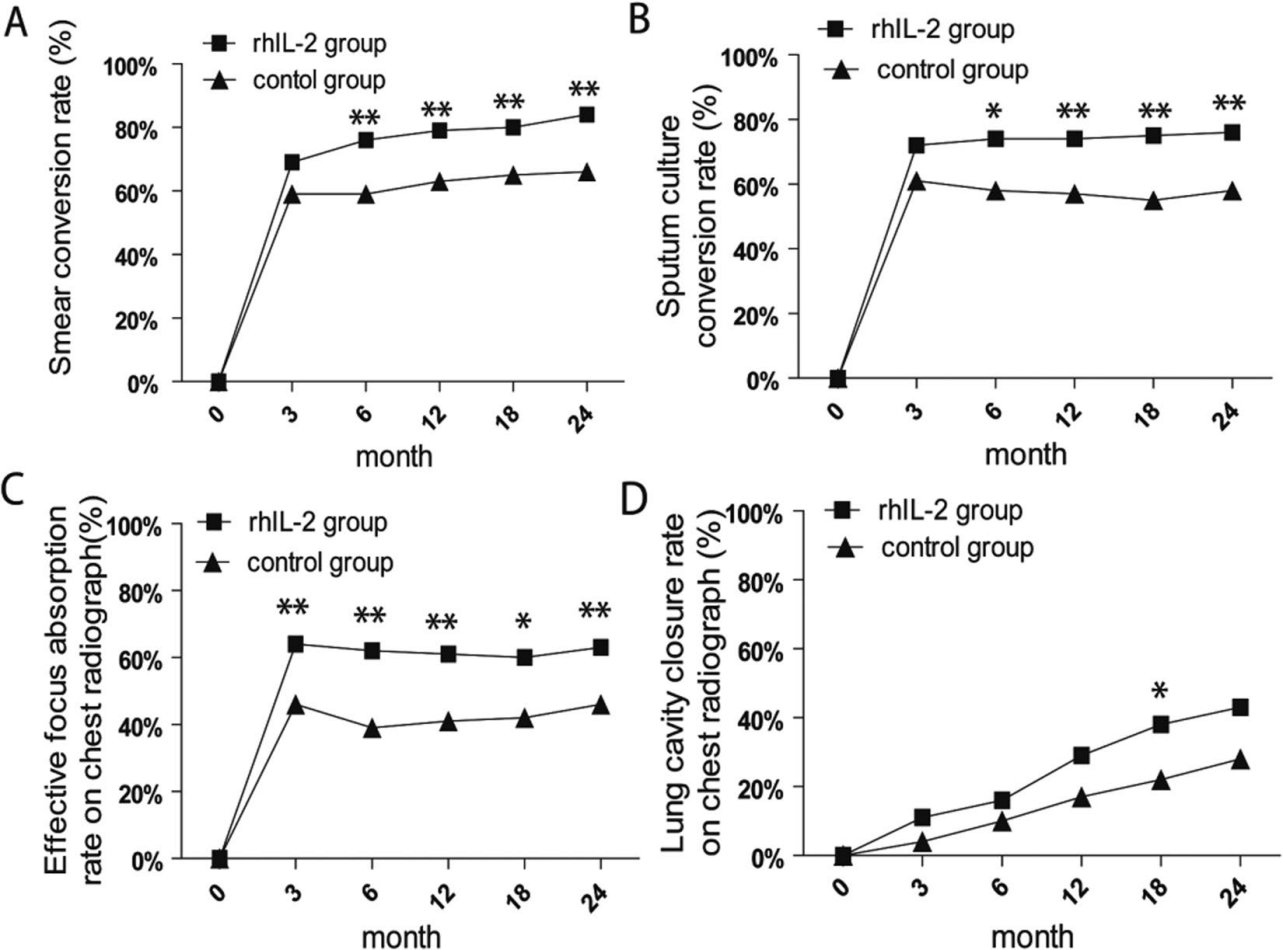
Higher rates of patients who achieved bacteriological sputum (smear and culture) conversion and radiological lung (focus and cavity) improvement in rhIL-2 group during follow-up months. Accumulated smear conversion rates (**A**), culture conversion rates (**B**), effective focus absorption rates observed on chest radiographs (**C**) and lung cavity closure rates observed on chest radiographs (**D**) at follow-up time points during treatment regimens for the two groups of MDR-TB patients. N (rhIL-2 group) = 117; N (control group) = 99. Data are shown with chi-squared (χ^2^) test or Mehta’s modification to Fisher’s exact test across the groups for comparisons of proportions (*p < 0.05, **p < 0.05).

Of 117 valid cases in rhIL-2 group, 80% achieved sputum smear conversion at the end of 18 month-treatment, notably higher in contrast to 65% of 99 valid cases in control group at the end of 24 month-treatment (*p* = 0.005, two-tailed), 75% achieved sputum culture conversion at the end of 18 month-treatment, notably higher in contrast to 57% of 99 valid cases in control group at the end of 24 month-treatment (*p* = 0.02, two-tailed). Proportions of cases achieved sputum culture conversion, effective focus absorption and lung cavity closure on chest radiographs in two groups were compared respectively at 3, 6, 12, 18, 24 months during follow-up (see Fig. 3). Cases of rhIL-2 group tended to perform greater improvement in shorter duration, compared to cases of control group at the end of regimen (18 months vs. 24 months) (Supplementary Table S3).

#### Immunologic effect

When participants enrolled, we randomly selected 25 study codes from the control treatment group and 25 study codes from rhIL-2 group with matched cases in 2 groups. Unfortunately, in rhIL-2 group, one case defaulted and the other one died, both not available for blood samples. Consequently, data from 25 cases in control group and 23 cases in rhIL-2 treatment were evaluated.

**Figure 4:**
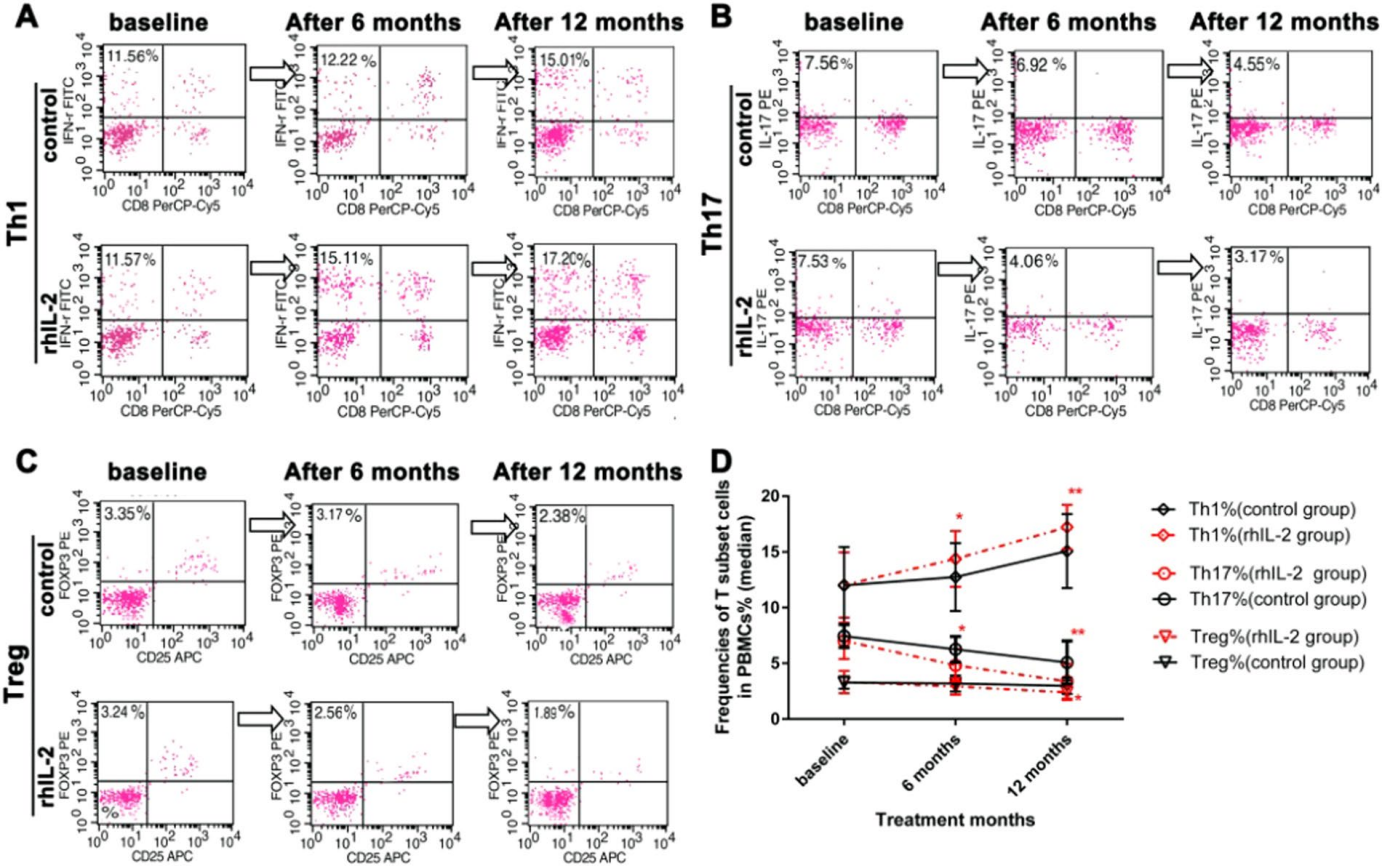
Increased frequencies of CD8^−^IFN-γ^+^cells in PBMCs of MDR-TB patients and decreased frequencies of IL-17A^+^CD8^−^cells, Foxp3^+^CD25^+^cells during and after rhIL-2 therapy. Flow cytometry measurements of T subset frequencies in PBMCs of patients from rhIL-2 group and control group with data from 25 cases in control group and 23 cases in rhIL-2 group were shown. Stopping and storage gates were set at 30,000 lymphocyte events defined by forward scatter (FSC) and side scatter.Numbers in plots indicate percent IL-17A^+^CD8^−^cells (top left) or CD8^−^IFN-γ^+^cells (top left) in the CD3^+^CD8^−^ gate, with isotype controls were used. For Treg cells staining PE-conjugated anti-human Foxp3 with isotype controls was used (BD Pharmingen™). Foxp3^+^CD25^+^cells (top right) in the CD4^+^CD25^+^ gate. Frequency of CD3^+^CD8^−^IFN-γ^+^T cells was higher in rhIL-2 group (**A**); frequency of CD3^+^CD8^−^IL-17^+^ cells was lower in rhIL-2 group (**B**); frequency of CD4^+^CD25^+^Foxp3^+^ T cells was lower in rhIL-2 group (**C**); as compared with the control group. Therapy with rhIL-2 facilitated the increasing trend of Th1 frequency, the decreasing trends of Th17 frequency and Treg frequency in the PBMCs from the patients in both groups(**D**). Data from 25 cases in control group and 23 cases in the rhIL-2 group were shown using student’s t test or one-way ANOVA with Tukey’s post-hoc test. All reported *p* values are two-tailed and unadjusted for multiple comparisons. (*p < 0.05, **p < 0.05).

Flow cytometry assay for T subsets measurement was shown in Fig. 4. Among participants who received rhIL-2 treatment, CD3^+^CD8^−^IFN-γ^+^ cells% were higher than those in control group at 6 month and 12 month. Among participants in both groups CD3^+^CD8^−^IFN-γ^+^% tended to increase during 12 month treatment compared to baseline. In contrast, among participants who received rhIL-2 treatment, CD3^+^CD8^−^IL-17^+^cells% and CD4^+^CD25^+^Foxp3^+^cells% were lower than those who received chemotherapy in control group at 6 month and 12 month, during 12 month treatment, among participants in both groups CD3^+^CD8^−^IL-17^+^cells% tended to decrease compared to baseline, CD4^+^CD25^+^Foxp3^+^cells% in IL-2 group tended to decrease compared to baseline. while CD4^+^CD25^+^Foxp3^+^ cells% in control group seemed no changes (Supplementary Table S4).

**Figure 5:**
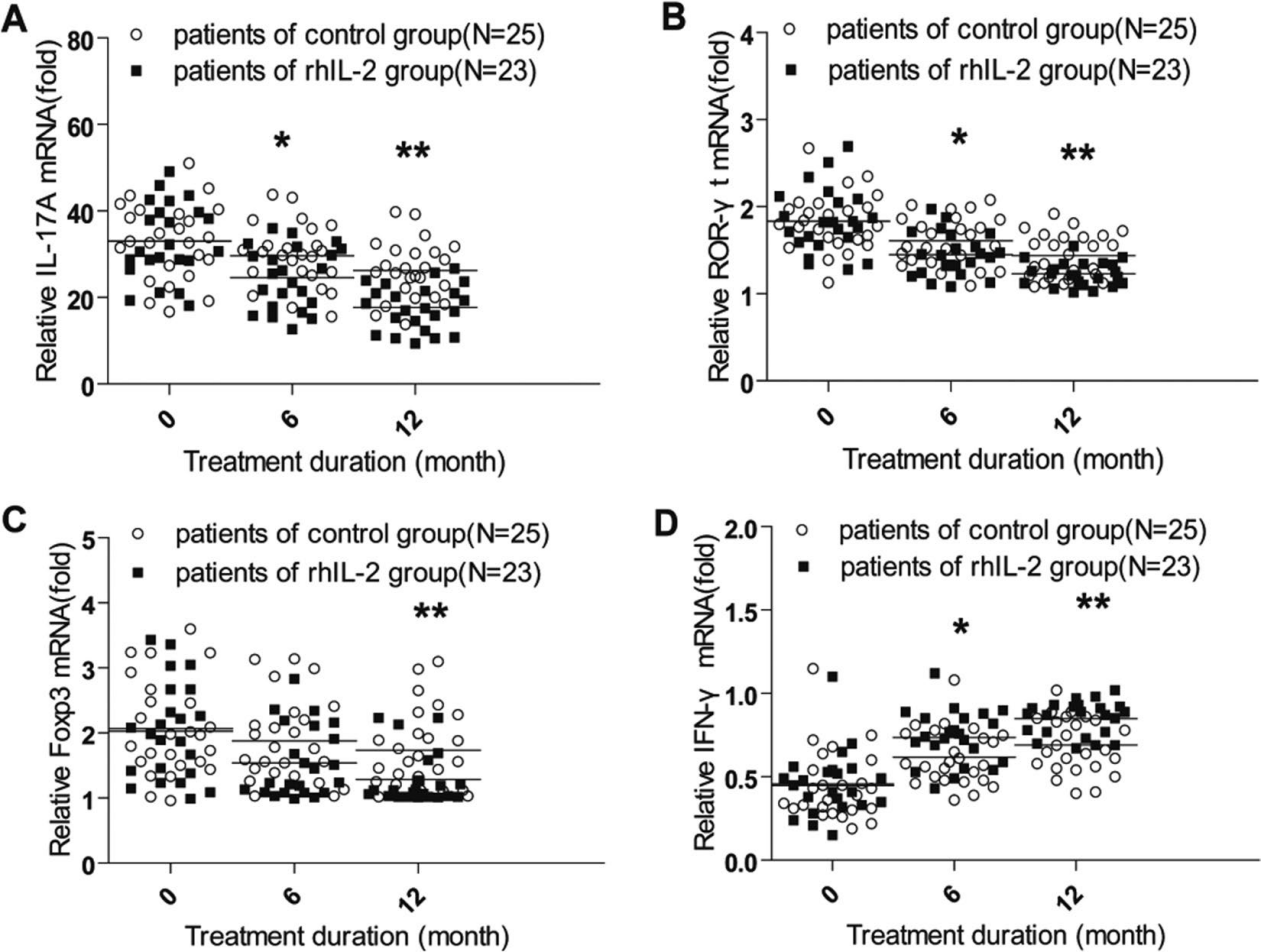
Increased level of IFN-γ mRNA, decreased levels of IL-17A, ROR-γt and FoxP3 mRNA in PBMCs from MDR-TB patients during and after rhIL-2 therapy. Quantitative Real-time PCR analysis of mRNA expression in patients from rhIL-2 group and control group with data from 25 cases in control group and 23 cases in rhIL-2 group were shown. Compared with patients in the control treatment group, patients receiving adjunctive rhIL-2 therapy had lower relative levels of IL-17A mRNA (**A**), ROR-γt mRNA (**B**), Foxp3 mRNA (**C**) and higher relative level of IFN-γ mRNA (**D**) in their PBMCs at the 6-month and 12-month time points during treatment regimens. Using student’s t test or one-way ANOVA with Tukey’s post-hoc test all reported *p* values are two-tailed and unadjusted for multiple comparisons. (*p < 0.05, **p < 0.05).

Realtime-qPCR assay for circulating mRNA levels of IL-17A, IFN-γ, ROR-γt, Foxp3 were shown in Fig. 5. at 6 month and 12 month during treatment rhIL-2 group cases performed distinctly higher IFN-γmRNA level than control group cases (*p* < 0.05, two-tailed). Among participants in both groups IFN-γmRNA level tended to increase during 12 month treatment compared to baseline (*p* < 0.05, two-tailed). In contrast, at 6 month and 12 month participants given rhIL-2 therapy performed lower IL-17A, ROR-γt mRNA. (*p* < 0.05, two-tailed). Among two groups of participants, IL-17A, ROR-γt mRNA levels tended to decrease compared to baseline (*p* < 0.05, two-tailed). Among rhIL-2 group cases, lower Foxp3 mRNA levels were detected compared to control group cases (*p* < 0.05, two-tailed). Foxp3 mRNA level in both group cases tended to decrease compared to baseline at 6 month and 12 month during treatment (*p* < 0.05, two-tailed). (Supplementary Table S5)

## Discussion

When we designed the trial to determine the clinical effect of rhIL-2 adjunctive immunotherapy in MDR-TB, few large-scale clinical trials had tested and compared the effect of a shorter course of immunotherapy regimen to a 24 month-course of standard chemotherapy regimen in MDR-TB patients. Johnson *et al*. in 1995 first reported the effect of rhIL-2 in seven patients with MDR-TB^23^. Then in 1997 Johnson *et al*. conducted another pilot study, totally about 20 MDR-TB patients were devided to 3 groups with treatment schedules induced different results, showing that proportion of patients receiving daily rhIL-2 therapy or 5 days followed by a 9-day ‘rest’, for three cycles pulse rhuIL-2 treated demonstrated reduced or cleared sputum bacterial load significantly^24^. In 1998 Johnson *et al*. found that daily rhIL-2 administration for 30 days induced increases in CD25^+^ and CD56^+^ T cells in the blood and in expression of IFN-γ and IL-2 mRNA at the site of a delayed-type hypersensitivity (DTH) response to purified protein derivative of tuberculin^25^. In 2003 a randomized controlled trial on re-treated Mycobacterium tuberculosis-positive pulmonary tuberculosis conducted by Chu NH, *et al*. reported that sputum smear-negative conversion rates and the X-ray resolution rates of cases(n = 103) with IL-2 plus chemotherapy during the first two month preceded over the control cases (n = 106)^26^. In 2003 Johnson *et al*. conducted another study of 101 cases cohorts IL-2 did not enhance bacillary clearance or improvement in symptoms in human immunodeficiency virus-seronegative adults with drug-susceptible tuberculosis^27^. These reports suggested a potential role for IL-2 in TB treatment and show mixed results of IL-2 treatment for TB.

Our study demonstrated that 8-month duration of rhIL-2 adjunctive therapy was safe and led to shortening of the traditional 24-month regimen by at least 6 months, with a striking increase in the cure rate from 37.2% to 55.6%. Consequently, the reduced toxicity of the shorter treatment course increased the tolerability and compliance rate of anti-TB chemotherapeutics. As for general trend of our study data, IL-2 immunotherapy accelerated clearance of intrapulmonary mycobacteria and effective tissue repair, thus accelerated clinical cure.

Although the mechanisms underlying the beneficial effect of rhIL-2 immunotherapy remained uncompletely understood, our previous work indicated that the responses of the Th1 and Th2 subsets in patients with MDR-TB are more substantially suppressed^13^, while Th17 subset response seemed more preferentially enhanced^15^, as it was reported that CD4^+^ T cells fail to produce IL-2 during chronic TB infections^11^. The use of supplemental IL-2 *in vitro* has been evidenced to restore some of the anti-bacterial reactivity of T cells^8–10^. It is thus possible that IL-2 therapy improved anti-TB immune responses and thereby had an effect on the bacterial load.

To elucidate the mechanisms underlying rhIL-2 effect *in vivo*, we observed activations of T subsets induced by rhIL-2 adjunctive treatment in these participants. We reported an restriction of CD3^+^CD8^−^IL-17^+^ cells generation and an expansion of CD3^+^CD8^−^IFN-γ^+^ T cells activation as well as limitation of CD4^+^CD25^+^Foxp3^+^cells population *in vivo* induced by adjunctive rhIL-2 immunotherapy. Interestingly, the trends of CD3^+^CD8^−^IFN-γ^+^cells and CD3^+^CD8^−^IL-17^+^cells populations were inversed during rhIL-2 immunotherapy as the clinical symptoms improved.

Recent studies have demonstrated that the balance between Th17-mediated protection and pathology is crucial for defining the outcome of infections at the mucosa with IL-17 attracting IFN-γ-producing CD4^+^ Th1 cells to the lungs to control the infection^19,20^. Our previous study suggested that chronic infection with MDR-TB probably exhausted Th1 cells and activated the Th17 cell response. As was reported, pathogen specific Th17 cells generated during mycobacterium infection induce the expression of CXCL9, CXCL10 and CXCL11, as well as IL-17 produced dictate the migration of other important effectors cell types to control the infection^28,29^. In our previous study we detected that MDR-TB patients more remarkably suppressed Th1 and Th2 cell response and more significantly enhanced IL-17 expression versus DS-TB according to our publications^13,15^, which are characterized in early infection of MDR-TB. From these evidence we obtained we supposed that higher Th17 frequency in MDR-TB manifested were potentially related to symptom severity especially in drug-resistant TB infection. We speculated the expansion of circulating Th17 cells in MDR-TB patients developed from three T cell populations. Firstly, the γδT cell population, which is recently elucidated to be a major and primary source of mycobacterium-induced IL-17 during mycobacterium infections^30–32^. Secondly, the conversion of Treg to Th17 cells has now been reported, in both mouse and human in an IL-1-dependent manner^33–35^. Induced Treg cells converted into IL-17-producing cells that maintained the expression of Foxp3, and give rise to ‘hybrid’ pro-inflammatory effectors in the context of an infectious challenge^36^. Thirdly, the impaired Th1 response induced the generation of Th17 and IL-17 to promote the clearance of micobacteria^19^.

IL-2 derived from lung DC cells was recently reported to induce protective Th17 response and restrain fatal hyperinflammation *in vivo* ^37^. IL-2 restrictes *in vitro* generation of IL-17-Secreting CD4^+^T cells via STATA-5, IL-2 and STAT5a/b, which were known to be key regulators of Treg cells, also serve to constrain Th17 polarization^38^. IL-2 can also act T cell intrinsically to dampen differentiation of pathogenic IL-17-producing Th17 cells^38^. It has been reported that tissue resident macrophage IL-2 drives IFN-γ and IL-27 production in CD11b + APC to dampen pathogenic Th17 differentiation^39,40^.

Notably in Th17 cells, Tbx21 retroviral transduction increases IFN-γ production, suggesting that IL-2 stimulation can promote a Th17-to-Th1 cell shift in these cells^40^. IL-2-induced IL-12Rβ2 is critical for Th1 cell differentiation^41^.

Plasticity of induced Treg cells and Th17 cells were indicated by epigenetic modifications of lineage-specifying transcription factors and cytokines^36^. As reported MTB infection is associated with an increase in the frequency of CD4^+^CD25^+^FoxP3^+^Treg in the blood and at the site of infection, resulting in MTB specific immunity suppression that may foster the chronicity of MTB infection^42^. However the role of Treg cell in the infection of TB is complicated as whether it is protective, deteriorated, or bystanding is still not clear.

Infection with M. tuberculosis may induce Treg cell-surface molecular changes with increased numbers of cell^42^. However, the mechanisms for Treg cells decreased in number after cure of active TB was not clear. On the one hand, the minimum dose of IL-2 necessary to stimulate Treg cells has not yet been established, and may vary according to the patient and the diseases. The main conclusions are that IL-2 induces robust expansion of the Treg cell population in mice and in patients with autoimmune vasculitis secondary to hepatitis C virus infection chronic^43^, graft-versus-host disease (GVHD)^44^. However on the other hand, it seemed that the decreased trend of Treg cells in these patients after IL-2 treatment overpowered the increasing trend induced by IL-2 itself. The bacterial burden may induce the increase in Treg cells and consequently as the burden released (mycobacterium clearance) Treg cells decreased, which was evidenced from the patients with cavity MDR-TB, showing higher proportion of CD4^+^CD25 high cells before treatment and CD4^+^CD25^+^FoxP3^+^cells was significantly decreased in peripheral blood at 6-months after surgery^45^.

Three limitations of this study should be noted. Firstly, our clinical outcomes analysis unbound cellular immunity data due to limited sample size for immunology test, while we found some patients of rhIL-2 group, who failed to benefit from immune treatment,. Stratified analyses according to cellular immune baseline in MDR-TB patients are needed in further study to prognosticate and select patients beneficial from rhIL-2 therapy. Secondly, selection bias due to limited sample size cannot be completely avoided; our data only represent the retreated MDR-TB categories. Thirdly, the present study was designed as an open-label trial unavoidable for comparing the compliance of MDR-TB patient under subcutaneous injection treatment as well as the effectiveness of rhIL-2 immunotherapy to intensive standard chemotherapy, which was not appropriate to a blind control study and consequently increasing biases.

In conclusion, the adjunctive rhIL-2 therapy in proved to be safe and conferred significant improvement in cure rate, earlier culture negativity and lung focus absorption in MDR-TB patients, with the standard chemotherapy duration potentially shortened for 6 months, and The results showed that rhIL-2 immunotherapy seemed to promote the conversion of the Th1/Th17 activation imbalance and limit the Treg activation *in vivo*. Our study ascertained a clue that rhIL-2 effectively improve the treatment for MDR-TB probably via restoring imbalance of CD4^+^ T cell activations and favorably modulating Th1/Th17 pathway response *in vivo*. In further study, stronger evidence for novel target immunotherapy with optimal dose of rhIL-2 immunotherapy, treatment duration as well as patients selection bias should be confirmed.

## Methods

### Study oversight

This prospective two arm-randomized controlled trial was conducted among 287 culture-comfirmed MDR-TB patients at The First Affiliated Hospital of Nanjing Medical University based on the network system established by the Centers for Disease Control (CDC) of Jiangsu Province for TB control. Totally 14 reference centres were located in 14 cities throughout eastern China (coordinating hospital specialized in tuberculosis listed in Supplementary Table S1) to screen and enroll qualified MDR-TB cases. Participants were enrolled and started their treatment from January 2009. Follow-up data of all participants were collected and analysed until July 2016 (last follow-up visit). The present study protocol was obtained from the ethics committee of the First Affiliated Hospital of Nanjing Medical University (approval number 2008-Ethical sequence-0802) and was performed adhering to the ethical principles of the Declaration of Helsinki. This study is registered with ClinicalTrials.gov (registration number NCT03069534, date of registration 02/27/2017). All patients provided written informed consent before enrollment. The independent data and safety of the study were monitored by the hospital monitoring committee.

### Patient population

Based on the multi-center collaboration, the required sample size for this study was calculated with the following formula: n = (*U*_*α*_ + *U*_*β*_)^2^2P(1-P)/(P_1_-P_0_)^2^. Each MDR-TB case was identified by triplicate-spot sputum smears that were positive for acid-fast bacilli and a positive sputum culture with resistance to both isoniazid and rifampin, as determined by susceptibility tests and rapid screening tests (BD BACTEC MGIT 960). The results of these assays were confirmed using an L-G medium base modified in Jiangsu province CDC. Enrollment criteria: Patients had/were: (1) a confirmed case of MDR-TB; (2) aged 18–70 years old; (3) a chest CT showing visible lung lesions, with or without holes; (4) a fasting plasma glucose of less than 7.8 mol/L and a normal fungus examination, if they were diabetic; (5) voluntarily joined this study and signed an informed consent form. Excluding criteria: (1) two or more total allergies or any drug or food allergies; (2) resistant to some drugs of this program; (3) severe disorders of liver, kidney, or hematologic system functions; (4) any metabolic diseases, autoimmune diseases, endocrine diseases, cancer, or HIV/AIDS; (5) a long-term use of immunosuppressive agents; (6) a blood system dysfunction; (7) a history of mental illness or epilepsy; (8) pregnant or lactating; (9) participated in another clinical trial in the last 3 months or were currently participating in other ongoing clinical trials; (10) long-term alcohol abuse >10 years and more than two alcoholic drinks per day); (11) any other factor rendering them unsuitable to participate in this project, such as a history of unreliability.

### Study design and treatment plan

**Figure 6:**
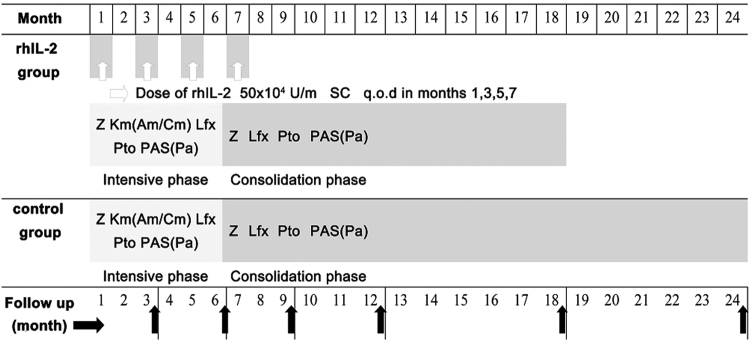
Treatment plan for the two cohorts. Patients in control group received an optimized anti-MDR-TB chemotherapy regimen. Patients in rhIL-2 group received an optimized anti-MDR-TB chemotherapy regimen combined with rhIL-2 therapy. Optimized anti-MDR-TB chemotherapy included a 6-month course of Z + KM/AM or CM + PAS/Pa + PTO) + LFX as an intensive phase treatment, followed by an 18-month course of Z + LFX + PTO + PAS/(Pa) as a consolidation phase treatment. PAS, PTO, LFX, and Z were administered orally once per day. KM/AM and CM were administered by intravenous injection once or twice per day according to patient tolerance. The 8-month treatment of rhIL-2 therapy included four courses of low-dose rhIL-2 (50 × 10^4^ U/m) given subcutaneously (SC) once every other day (q.o.d.) for 30 days, with four courses carried out separately during months 1, 3, 5, and 7. Z: Pyrazinamide; KM: Kanamycin; AM: Amikacin; CM: Capreomycin; LFX: Levofloxacin; PTO: Prothionamide; PAS: Para-aminosalicylicacid; Pa: Pasiniazid; rhIL-2: Recombinant human Interlukine-2; all of the agents were produced domestical.

Study consort flow diagram see Fig. 1. Randomization was performed at a 1:1 ratio and each patient was assigned a unique study number selected sequentially that were stratified according to the study center. The study used concealed allocation through an interactive voice response system that centrally assigned. Each enrolled patient was assigned the next available study code that corresponded to a prepackaged bottle of study drug. Clinical data were captured on structured case report forms that we reentered into a secure Web-base database. Eligible patients were assigned into one of the following regimens: 1.control regimen, to receive the background drug regimen; 2. rhIL-2 regimen, to receive the background drug regime plus rhIL-2 therapy. Optimized anti-MDR-TB chemotherapy regimen, given as control regimen, with a 6-month course intensive phase treatment, followed by an 18-month course of consolidation phase treatment, was developed according to the World Health Organization guidelines for MDR-TB treatment^46,47^. Modifications to this background regimen were allowed because of unacceptable adverse events. Intake of all study medications was supervised to ensure adherence by the patients. Drug doses were adjusted according to patient weight (Supplementary Table S6). The rhIL-2 therapy regimen over a period of 8 months consisted of four courses of low-dose rhIL-2 (50 × 10^4^ U/m) given subcutaneously once every other day (q.o.d.) for 30 days separately during months 1, 3, 5, and 7(Fig. 6).

### Study outcomes

The treatment outcome definitions were adapted from WHO Report on Global Tuberculosis Control and the WHO guidelines for the programmatic management of drug-resistant TB(Supplemental Methods. Treatment outcome definitions). The primary outcome was the proportion of patients who had clinically defined cure and treatment success within 18 months of follow duration. Secondary outcomes were the proportion of patients who achieved bacteriological sputum (smear and culture) conversion or radiological lung focus (cavity) improvement at the end of treatment. The safety outcome was the proportion of patients with adverse events (AE) or adverse reactions that were graded according to a modified version of Common Terminology Criteria for Adverse Events v4.0 (CTCAE).

### Clinical monitoring

The proportions of MDR-TB patients in the two groups achieving sputum smear conversion, sputum culture conversion, radiological lung focus (cavity) improvement were obtained and compared at follow-up months 3, 6, 9, 12, 18, and 24. Sputum was decontaminated with acetylcysteine–sodium hydroxide, examined microscopically, and cultured on Lowenstein–Jensen solid medium and in liquid medium in a Mycobacterium Growth Indicator Tube (MGIT) (Becton Dickinson). Safety monitoring for rhIL-2 and chemotherapeutics, which included clinical symptom observation, physical examinations, and clinical laboratory tests, were performed monthly during follow-up until 24 months. Incidences of AE in two groups were assessed and compared. Clinical symptom observation included cough, expectoration, fever, fatigue, weight, chest tightness, hemoptysis and appetite etc. Physical examinations included vital signs. Clinical laboratory tests included blood routine test, hematologic profile, blood biochemical examination including hepatic aminotransferase and blood ureantrogen/creatinine, hepatitis B antigen/antibody two-and-half assay before treatment, blood electrolyte test (potassium, magnesium, calcium), serum thyroid stimulating hormone (TSH), audiology examinations, visual field and color examination, standard 12-lead electrocardiography, if required. Clinical effect assessment included microbiologic assessment, imaging assessment, safety assessment.

### Immune assessment

T cell status was measured in 25 patients in the rhIL-2 group and 25 patients in the control group to assessment immune effect. these patients were randomly selected with matched locations (samples transferring and restoring) and enrolled seasons (outdoor temperature). Flow cytometry and real-time quantitative PCR (RT-qPCR) were used to assess the cellular and mRNA levels in these patients from two groups, at baseline and at 6 and 12 follow-up months.

#### *Sample preparation*

In the morning, 5 ml of peripheral blood was drawn by venipuncture aseptically from the median basilica vein of each participant.

#### *Isolation of PBMCs*

The PBMCs were isolated from the 5 ml blood samples using Ficoll-Paque Plus (GE, USA). The PBMCs from each BCT were always brought to a concentration of 2 × 10^6^ cells per system for further *in vitro* culture.

#### *In vitro* culture

For the measurement of Th1 and Th17 cells, the PBMC suspensions in each well were stimulated and cultured for 6 hours with 4 µl of Leukocyte Activation Cocktail (BD Pharmingen™, CA, USA).

#### *Determination of cell phenotype by flow cytometry*

PBMCs harvested were stained with APC-conjugated anti-CD3 and PERCP-CY5.0-conjugated anti-CD8 at room temperature. APC-mouse IgG1 and PERCP-CY5.0-mouse IgG1 (Immunotech) were used as isotype controls. For intracellular staining, FITC-conjugated anti-human IFN-γ and PE-conjugated anti-human IL-17 were used with FITC-mouse IgG1 and PERCP-CY5.5-mouse IgG1 (Immunotech) used as isotype controls. For Treg cells staining PE-conjugated anti-human Foxp3 with isotype controls was used (BD Pharmingen™). For flow cytometry analysis, stopping and storage gates were set at 30,000 lymphocyte events defined by forward scatter (FSC) and side scatter. The analysis included identification of the percentage of positive cells CD3^+^CD8^−^IFN-γ^+^, CD3^+^CD8^−^ IL-17^+^, CD4^+^CD25^+^ Foxp3^+^ as Th1, Th17, Treg cells. Files were exported in Flow Cytometry Becton Dickinson FACSCalibur and visualised using BD Cell Quest Pro Software.

#### *Determination of cytokine mRNAs by real-time quantitative PCR (qRT-PCR)*

For the determination for IFN-γ, IL-17A, ROR-γt, and Foxp3 mRNA, RNA was extracted from PBMC by using TRIZOL Reagent® according to the manufacturer’s protocol (Invitrogen, USA). Then, 500 ng of total RNA was reverse transcribed in a final volume of 10 μ l using random primers and standard conditions with the Prime Script RT Master Mix (Takara, Cat. #RR036A) as cDNA synthesis was performed. Quantitative quantitative real-time polymerase chain reaction (qRT-PCR) was performed with SYBR Select Master Mix (Applied Biosystems, cat:4472908) on ABI System Applied Biosystems StepOne™ Real-Time PCR System according to the manufacturer’s instructions. We used β-action as internal controls. We used RNA samples from healthy donors as negative control. Target mRNA (IFN-γ, IL-17A, Foxp3, and ROR-γt) primers design and sequence tested for qRT-PCR amplification were summarized in Supplementary Table S7. We performed 3-step assays on mRNA expression (β-action and Target mRNA). The qRT-PCR reaction included an initial denaturation step at 95 °C for 10 min, which was then followed by 40 cycles of 92 °C for 15 s and 55 °C (annealing temperature) for 1 min. We used the ΔΔ Ct method to determine expression fold changes (patients vs. normal) in subsequent calculations. Conditions of qRT-PCR amplification system were summarized in Supplementary Table S8.

### Statistical Analysis

All statistical tests in this study were performed using SPSS 17.0 software (SPSS Inc., Chicago, IL, USA). The data were expressed as the mean ± standard deviation. The clinical characteristics of the rhIL-2 group and the control group were compared using Levene’s variance test of homogeneity. We used the chi-squared (χ^2^) test or Mehta’s modification to Fisher’s exact test across the treatment groups for comparisons of proportions. We compared Th17, Treg, and Th1 cell frequencies and the relative levels of IL-17A, ROR-γt, Foxp3, and IFN-γ mRNA in PBMCs among all groups at baseline and between the two treatment groups at 6-month and 12-month follow-ups using Student’s t test or one-way ANOVA with Tukey’s post-hoc test. All reported *p* values are two-tailed and unadjusted for multiple comparisons.

## Electronic supplementary material

SUPPLEMENTARY INFO
