# Supplementary material for: Clinical and Immunological Effects of rhIL-2 Therapy in Eastern Chinese Patients with Multidrug-resistant Tuberculosis
Source: Sci Rep. 2017 Dec 19;7:17854. doi: 10.1038/s41598-017-18200-5 (PMC5736576; doi:10.1038/s41598-017-18200-5)
Supplement: Supplementary file 1 — SUPPLEMENTARY INFO [file 41598_2017_18200_MOESM1_ESM.doc]

**Clinical and Immunological Effects of rhIL-2 Therapy in Eastern Chinese Patients with Multidrug-resistant** _Tuberculosis_

**Qi Tan^1*^, Rui Min^1*^, Guan-qun Dai^1^, Yan-li Wang^1^, Li Nan^1^, Zhen Yang^2^, Jun Xia^3^, Shi-yang Pan^4^, Huang Mao^1^, Wei-ping Xie ^1**^ & Hong Wang ^1**^**

^1^Department of Respiratory and Critical Care Medicine, the First Affiliated Hospital of Nanjing Medical University, Nanjing, 210029, China. ^2^Jiangbei Hospital, Nanjing, 210029, China.  ^3^Jiangsu Province Hospital of TCM, the Affiliated Hospital of Nanjing University of TCM, Nanjing, 210029, China. ^4^Department of Laboratory Medicine, the First Affiliated Hospital of Nanjing Medical University, Nanjing, China. *These authors contributed equally to this work. **Correspondence and requests for materials should be addressed to H.W. (email: [hongwangnjmu@hotmail.com](mailto:hongwangnjmu@hotmail.com)) or W.-P. X. (email: wpxie@njmu.edu.cn）

**Supplementary Information**

| **Sub-centers** | **Enrolling cases** | **RhIL-2 group(n)** | **Control group(n)** |
| --- | --- | --- | --- |
| LYG Hospital | 27 | 16 | 11 |
| XZ Hospital | 30 | 17 | 13 |
| NT Hospital | 28 | 20 | 8 |
| YZ Hospital | 16 | 10 | 6 |
| ZJ Hospital | 52 | 13 | 39 |
| CZ Hospital | 19 | 12 | 7 |
| SZ Hospital | 17 | 14 | 3 |
| NC Hospital | 38 | 18 | 20 |
| TZ Hospital | 30 | 19 | 11 |
| TX CDC | 2 | 2 | 0 |
| HA Hospital | 10 | 7 | 3 |
| SQ CID | 10 | 6 | 4 |
| WX Hospital | 8 | 5 | 3 |
| YC Hospital | 15 | 10 | 5 |
| **SUM** | **302** | **169** | **133** |

**Supplementary Table S1. Sub-centers and enrollment.** Cases enrollment in two cohorts separately from 14 sub-centers were shown in table. LYG Hospital, The Forth Hospital of Lianyungang City. XZ Hospital, Xuzhou Infectious disease hospital. NT Hospital, The Sixth Hospital of Nantong City, YZ Hospital. The Third Hospital of Yangzhou City. ZJ Hospital, The Third Hospital of Zhenjiang City. CZ Hospital, The Fifth Hospital of Changzhou City. SZ Hospital, The Fifth Hospital of Suzhou City. NC Hospital, Nanjing Chest Hospital. TZ Hospital, The First Hospital of Taizhou City. TX CDC, Center for Disease Control and Prevention (CDC) of Taixing City. HA Hospital, The Forth Hospital of Huai’an City. SQ Hospital, Center for infectious-disease control (CID) of Suqian City. WX Hospital, Infectious disease hospital of Wuxi City. YC Hospital, The Second Hospital of Yancheng City.

|  | **RhIL-2 group （18month）** | **Rate**  **(%)** | **Control group （24month）** | **Rate**  **(%)** | **Comparement between two groups** | |
| --- | --- | --- | --- | --- | --- | --- |
| **Enrollment** | **N=142** |  | **N=129** |  | **χ^2^value** | **P value**  **(*P<0.05)** |
| **Cure** | **79** | **55.6%** | **48** | **37.2%** | **8.49** | **0.003*** |
| **Completion** | **20** | **14.1%** | **25** | **19.4%** | **1.01** | **0.31** |
| **Success**  **(Cure+ Completion)** | **99** | **69.7%** | **73** | **56.6%** | **4.47** | **0.034*** |
| **Failure** | **18** | **12.7%** | **26** | **20.2%** | **2.26** | **0.13** |
| **Death** | **5** | **3.5%** | **7** | **5.4%** | **0.47** | **0.64** |
| **Dropped out** | **20** | **14.1%** | **23** | **17.8%** | **0.68** | **0.50** |

**Supplementary Table S2. Outcome comparison of patients between the two groups.** The data was analyzed by chi–square test or fish’s exact test. **p value* < 0.05 ( control group vs. rhIL-2 group).

| **Follow-up months** | **Sputum and imaging assessment** | **rhIL-2 Group** | **Contol group** | **χ^2^ value** | **P value** |
| --- | --- | --- | --- | --- | --- |
| 3 month | Smear conversion(N=117/99) | 81/117（69.2%） | 59/99 （59.6%） | 0.78 | 0.38 |
|  | Culture conversion(N=117/99) | 86/117（71.8%） | 58/99（60.0%） | 3.02 | 0.08 |
|  | Lung focus absorbtion(N=117/99) | 75/117 (64.1%） | 46/99（46.5%） | 6.77 | 0.009 |
|  | Lung cavities closure(N=95/78) | 11/95（11.6%） | 3/78（3.8%） | 3.44 | 0.06 |
| 6 month | Smear conversion(N=117/99) | 89/117 (76.1%） | 59/99 (59.6%） | 6.75 | 0.009* |
|  | Culture conversion(N=117/99) | 86/117（73.5%） | 58/99（58.6%） | 5.37 | 0.02* |
|  | Lung focus absorbtion(N=117/99) | 73/117 (62.4%） | 39/99 (39.4 %） | 11.36 | <0.001 |
|  | Lung cavities closure(N=95/78) | 15/95（15.8%） | 8/78（10.2%） | 1.14 | 0.28 |
| 12month | Smear conversion(N=117/99) | 93/117 (76.5%） | 63/99(63.6%） | 6.72 | 0.009* |
|  | Culture conversion(N=117/99) | 86/117（73.5%） | 56/99（56.6%） | 6.83 | 0.009* |
|  | Lung focus absorbtion(N=117/99) | 71/117（60.7%） | 41/99（41.4%） | 7.98 | 0.005* |
|  | Lung cavities closure(N=95/78) | 28/95（29.5%） | 13/78（16.7%） | 3.21 | 0.07 |
| 18month | Smear conversion(N=117/99) | 94/117 (80.3%) | 64/99 (64.6%) | 6.73 | 0.009* |
|  | Culture conversion(N=117/99) | 88/117（75.2%） | 55/99（55.6%） | 9.26 | 0.002* |
|  | Lung focus absorbtion(N=117/99) | 70/117（59.8%） | 42/99（42.4%） | 6.51 | 0.011 |
|  | Lung cavities closure(N=95/78) | 36/95（37.9%） | 17/78（21.8%） | 4.49 | 0.03 |
| 24month | Smear conversion(N=117/99) | 98/117 (83.7%) | 65/99 (65.6%) | 9.49 | 0.002* |
|  | Culture conversion(N=50/51) | 89/117 (76.1%) | 57/99 (57.6%) | 8.37 | 0.004* |
|  | Lung focus absorbtion(N=117/99) | 74/117 (63.2%) | 46/99 (46.5%) | 6.12 | 0.0013 |
|  | Lung cavities closure(N=95/78) | 41/95（43.2%） | 22/78（28.2%） | 3.52 | 0.06 |
| End of regimen  (18month vs 24month) | Smear conversion(N=117/99) | Rate of 18 month | Rate of 24 month | 5.22 | 0.02* |
|  | Culture conversion(N=117/99) | Rate of 18 month | Rate of 24 month | 6.78 | 0.01* |
|  | Lung focus absorbtion(N=117/99) | Rate of 18 month | Rate of 24 month | 3.33 | 0.07 |
|  | Lung cavities closure(N=95/78) | Rate of 18 month | Rate of 24 month | 1.40 | 0.23 |

**Supplementary Table S3.**  **Comparison of proportions of patients achieving sputum smear/culture conversion and lung focus/cavity improvement on chest radiograph between the two groups at all followed up time points during treatment regime (accumulate).** **p value* < 0.05 (control group vs. rhIL-2 group)., N(rhIL-2 group)=117. N(control group)=99. Of 117 cases in rhIL-2 group, 95 cases with pulmonary cavities were confirmed by chest X ray when enrolled. Of 99 cases in control group, 78 cases with pulmonary cavities were confirmed by chest X ray when enrolled.

| **Subjects** | **Patients of**  **Control group** | **Patients of**  **rhIL-2 group** | **Homogeneity of variance test**  **P value** | **Student’s t test/ Chi-square test**  **value** |
| --- | --- | --- | --- | --- |
| **Subjects selected** | **25** | **25** |  |  |
| **Death or default** | **/** | **2** |  |  |
| **Subjects evaluated** | **25** | **23** |  |  |
| **Age (years)** | **45.93±11.51** | **46.12±12.17** | **0.96** | **t=0.06** |
| **Mean±SD** |
| **Gender** | **13(52%)**  **12(48%)** | **13(57%)**  **10(43%)** | **0.72** | **χ^2=^0.12** |
| **Male**  **Female** |

**Initial sampling time 2010.December-2011.Feburary**

| **CD4+T cells（%）** | **months** | **Patients of Control group(N=25)** | **Patients of rhIL-2 group(N=23)** | ***P* value** |
| --- | --- | --- | --- | --- |
| CD3^+^CD8^-^IL-17^+^cells | baseline | 7.45±1.03 | 7.02±1.63 | 0.52 |
|  | 6 | 6.25±1.15 | 4.83±1.46 | 0.02* |
|  | 12 | 5.07±1.94 | 3.38±1.56 | <0.01* |
| CD4^+^CD25^+^Foxp3^+^cells | baseline | 3.28±0.55 | 3.32±0.99 | 0.88 |
|  | 6 | 3.19±0.72 | 2.92±0.73 | 0.21 |
|  | 12 | 2.98±0.73 | 2.39±0.66 | <0.01* |
| CD3^+^CD8^-^IFN-γ^+^ cells | baseline | 11.99±3.46 | 12.03±2.93 | 0.92 |
|  | 6 | 12.73±3.05 | 14.36±2.50 | 0.04* |
|  | 12 | 15.07±3.32 | 17.23±1.99 | 0.01* |

**Supplementary Table S4. Comparison of proportions of CD3^+^CD8^-^IL-17^+^cells, CD4^+^CD25^+^Foxp3^+^cells, CD3^+^CD8^-^IFN-γ^+^ cells（%）between patients from IL-2 group and patients from control group at 0, 6, 12 months during treatment regime.** Data are means ±SD. **p value* < 0.05 (patients with control regimen vs. patients with rhIL-2 regimen). N(control regimen)=25, N(rhIL-2 regimen)=23.

| **relative mRNA levels（fold）** | **months** | **Patients of Control regimen(N=25)** | **Patients with rhIL-2 regimen(N=23)** | **P value** |
| --- | --- | --- | --- | --- |
| IL-17A mRNA | baseline | 32.93±8.79 | 33.02±8.83 | 0.97 |
|  | 6 | 29.60±7.24 | 24.57±7.09 | 0.02* |
|  | 12 | 26.61±6.64 | 17.68±5.24 | <0.01* |
| ROR-γt mRNA | baseline | 1.83±0.33 | 1.84±0.37 | 0.96 |
|  | 6 | 1.61±0.28 | 1.45±0.24 | 0.04* |
|  | 12 | 1.44±0.26 | 1.23±0.14 | <0.01* |
| IFN-γ mRNA | baseline | 0.45±0.21 | 0.46±0.20 | 0.87 |
|  | 6 | 0.62±0.17 | 0.74±0.16 | 0.02* |
|  | 12 | 0.69±0.17 | 0.85±0.10 | <0.01* |
| Foxp3 mRNA | baseline | 2.07±0.73 | 2.03±0.74 | 0.83 |
|  | 6 | 1.88±0.65 | 1.54±0.55 | 0.06 |
|  | 12 | 1.74±0.60 | 1.28±0.40 | <0.01* |

**Supplementary Table S5. Comparison of relative mRNA levels（fold）expression between patients from IL-2 group and patients from control group at 0, 6, 12 months during treatment regime.** Data are means ±SD. **p value* < 0.05 (patients with control regimen vs. patients with rhIL-2 regimen). N( control regimen)=25, N(rhIL-2 regimen)=23.

| **Agents** | **Dosage form** | **Dosage according to weight** | | | |
| --- | --- | --- | --- | --- | --- |
|  |  | **<33kg** | **33–50kg** | **51–70kg** | **>70kg** |
| Z | 500mg/tab | 25mg/kg/d | 750-1250mg/d | 1250-1750mg/d | 1750-2000mg/d |
| KM | 1g/bott | 15mg/kg/d | 500-750mg/d | 750-1000mg/d | 1000mg/d |
| AM | 0.2g/bott | 8mg/kg/d | 200-400mg/d | 200-400mg/d | 200-400mg/d |
| CM | 1g/bott | 0.5gd | 0.5-0.75g/d | 1g/d | 1g/d |
| LFX | 250mg | 750 mg | 750 mg | 750 mg | 750-1000 mg |
| PTO | 250mg/tab | 15mg/kg/d | 500mg/d | 750mg/d | 750-1000mg/d |
| PAS | 4g/bag | 4g/d | 8g/d | 8g/d | 8g/d |
| Pa | 0.1g/capsule | 0.6/d | 1.0g/d | 1.2 g/d | 1.2g/d |
| rhIL-2 | 0.5million  IU/bot | 0.25 millionIU/d | 0.5 millionIU/d | 0.5 millionIU/d | 0.5 millionIU/d |

1. **Supplementary Table S6.** **The doses of agents used in optimised anti-MDR-TB chemotherapy regimens.** Z: Pyrazinamide; KM: Kanamycin; AM: Amikacin; CM: Capreomycin; LFX: Levofloxacin; PTO: Prothionamide; PAS: Para-aminosalicylicacid; Pa:Pasiniazid; rhIL-2:Recombinant human Interlukine-2; all of the agents were produced domestical.
2. **Supplementary Methods**
3. **Treatment outcome definitions**
4. Treatment outcome definitions were adapted from WHO Report 2012 Global Tuberculosis Control and WHO guidelines for the programmatic management of drug-resistant TB:

*Cure:* Completion of treatment and at least five consecutive negative sputum cultures from samples collected at least 30 days apart or one positive sputum culture followed by a minimum of three consecutive negative cultures taken at least 30 days apart during the final 12 months of treatment;

*Treatment completion:* Completion of treatment not yet meeting the criteria for either cure or failure, with less than five consecutive negative sputum cultures recorded and a lack of microbiologic test results; without qualifying as either being cured or showing treatment failure,

*Treatment success: Cured or treatment completion;*

*Relapse:* Cure or treatment completion with at least one positive culture during post-treatment follow-up, unless the strain was proven to be different from the initial isolate by molecular techniques;

*Death:* Death from any cause during treatment.

The primary outcome was the cure rate and the secondary outcome was the assessment of a possible shortening of the treatment duration for MDR-TB patients who had rhIL-2 treatment added to their standard drug regimen for MDR-TB.

| **Primers** | **Primer sequence** | **Product length** |
| --- | --- | --- |
| Human IFN-γ-F | GCATCCAAAAGAGTGTGGAG | 235bp |
| Human IFN-γ-R | GCAGGCAGGACAACCATTAC |
| Human Foxp3-F | GAAACAGCACATTCCCAGAGTTC | 99bp |
| Human Foxp3-R | ATGGCCCAGCGGATGAG |
| Human IL-17-F | ACCAATCCCAAAAGGTCCTC | 170bp |
| Human IL-17-R | GGGGACAGAGTTCATGTGGT |
| Human RORγt -F | ACCTCACCGAGGCCATTCAG | 121bp |
| Human RORγt -R | TAGGCCCGGCACATCCTAAC |

1. **Supplementary Table S7. Target mRNA primers design and sequence tested for qRT-PCR amplification.**

| **Amplification system** | **Volume** |
| --- | --- |
| 2*SYBR Green Mix | 10ul |
| primer-F  primer-R | 0.35ul  0.35ul |
| cDNA template | 0.8-1ul |
| ddH2O | Add to 20ul |
| totel | 20ul |

**Supplementary Table S8. Conditions of qRT-PCR amplification system.**
